# Evaluation of platinum-free interval and chemotherapeutic effect of subsequent platinum-containing chemotherapy in patients with recurrent ovarian cancer initially treated with bevacizumab: SGSG018/Intergroup study

**DOI:** 10.1016/j.gore.2025.101740

**Published:** 2025-04-09

**Authors:** Tamaki Tanaka, Kazuhiro Takehara, Tomoka Usami, Masako Ishikawa, Eiji Kondo, Masahiro Kagabu, Kei Hirabayashi, Noriomi Matsumura, Shinya Sato, Masato Nishimura, Atsushi Arakawa, Keiichiro Nakamura, Yosuke Konno, Satoe Fujiwara, Kotaro Sueoka, Hiroko Nakamura, Iemasa Koh, Kimihiko Ito, Atsushi Hongo

**Affiliations:** a Department of Perinatology and Gynecology, Kagawa University Graduate School of Medicine, Kagawa, Japan; b Department of Gynecologic Oncology, NHO Shikoku Cancer Center, Ehime, Japan; c Department of Obstetrics and Gynecology, Ehime University Graduate School of Medicine, Ehime, Japan; d Department of Obstetrics and Gynecology, Shimane University Faculty of Medicine, Shimane, Japan; e Department of Obstetrics and Gynecology, Mie University Graduate School of Medicine, Mie, Japan; f Department of Obstetrics and Gynecology, Iwate Medical University, Iwate, Japan; g Department of Obstetrics and Gynecology, JCHO Tokuyama Central Hospital, Yamaguchi, Japan; h Department of Obstetrics and Gynecology, Kindai University Faculty of Medicine, Osaka, Japan; i Department of Obstetrics and Gynecology, Faculty of Medicine Tottori University, Tottori, Japan; j Department of Obstetrics and Gynecology, Tokushima Prefectural Central Hospital, Tokushima, Japan; k Department of Obstetrics and Gynecology, Nagoya City University West Medical Center, Aichi, Japan; l Department of Obstetrics and Gynecology, Okayama University Graduate School of Medicine, Dentistry and Pharmaceutical Sciences, Okayama, Japan; m Department of Obstetrics and Gynecology, Hokkaido University Hospital, Hokkaido, Japan; n Department of Obstetrics and Gynecology, Osaka Medical and Pharmaceutical University, Osaka, Japan; o Department of Obstetrics and Gynecology, Yamaguchi University Graduate School of Medicine, Yamaguchi, Japan; p Department of Obstetrics and Gynecology, NHO Kure Medical Center and Chugoku Cancer Center, Hiroshima, Japan; q Department of Obstetrics and Gynecology, Graduate School of Biomedical Sciences, Hiroshima University, Hiroshima, Japan; r Department of Obstetrics and Gynecology, Kansai Rosai Hospital, Hyogo, Japan; s Department of Obstetrics and Gynecology, Kawasaki Medical School, Okayama, Japan

**Keywords:** Ovarian cancer, Bevacizumab, Chemotherapy, Platinum-sensitive relapse, Platinum-free interval

## Abstract

**Objective:**

The effect of bevacizumab on platinum sensitivity in recurrent ovarian cancer remains poorly understood. This study examined the association between platinum-free interval (PFI) and sensitivity to subsequent platinum-containing chemotherapy in patients with first relapsed ovarian cancer after bevacizumab chemotherapy.

**Methods:**

We retrospectively analyzed patients who received platinum-based chemotherapy for platinum-sensitive recurrence between November 2013, and December 2019, and who were initially treated by platinum-based chemotherapy with concurrent and maintenance bevacizumab. The primary endpoint was response rate to subsequent chemotherapy after various periods of PFI. The relevance between response rate and PFI was assessed for each PFI of 6–12, 12–24 and ≧24 months using Cochran-Armitage test. The secondary endpoint was progression-free survival (PFS) defined as time from chemotherapy for first recurrence to subsequent progression and response rate to subsequent chemotherapy for each treatment-free interval since last administration of bevacizumab (Bev-TFI).

**Results:**

A total of 77 patients were eligible. The median PFI until first recurrence was 12 months (range: 6–43). The response rates of subsequent chemotherapy for patients with PFI of 6–12, ≥12-24, and 24 months were 42 %, 65 %, and 80 %, showing a linear trend (*p* < 0.05). Median PFS among the three groups was 8 (95 %CI: 6.7–9.2), 11 (95 %CI: 8.4–13.5) and 13 months (95 % CI: 5.4–20.5) (*p* = 0.107, log-rank test), respectively. By contrast, no linear trend was observed between Bev-TFI and response rate (*p* = 0.225)

**Conclusion:**

In patients with first relapse of primary ovarian cancer and bevacizumab beyond progression, the prolonged PFS effect of bevacizumab does not seem to affect sensitivity to subsequent platinum-based chemotherapy.

## Highlights

• The relationship between Platinum-free interval and response to subsequent platinum-based therapy did not change upon bevacizumab treatment.
• There was no correlation between treatment-free interval since last administration of bevacizumab and response rate for relapse treatment.
• The response rate was maintained even if relapse occurred during bevacizumab maintenance.

## 1 Introduction

Even if patients with stage III/IV advanced ovarian cancer achieve complete clinical remission through surgery and chemotherapy, more than 70 % of cases recur within 3 years (Ledermann et al., 2013). Additionally, the duration of response to recurrence treatment typically does not exceed that of initial chemotherapy (Harrison et al., 2007). Therefore, chemotherapy for recurrent ovarian cancer is performed to extend survival time and alleviate symptoms; however, long-term remissions after relapse treatment sometimes occur.

There have been several reports that the platinum-free interval (PFI), which is the period between the last administration of a platinum agent in pretreatment and the start of recurrence treatment, tends to correlate to subsequent sensitivity to platinum-based chemotherapy (Pujade-Lauraine, 2012, Gore et al., 1990, Colombo and Gore, 2007, Blackledge et al., 1989, Raja et al., 2013, Dizon et al., 2003, Markman et al., 1991). A PFI of < 6 months indicates platinum resistance, 6–12 months indicates partial sensitivity, and ˃12 months indicates sensitivity (Pujade-Lauraine, 2012). If the PFI is ≥ 6, platinum drugs are expected to be effective; therefore, combination therapy, including platinum drugs, is considered (Colombo and Gore, 2007, Raja et al., 2013, Dizon et al., 2003).

Currently, either or both bevacizumab and poly ADP-ribose polymerase (PARP) inhibitors are commonly combined with cytotoxic drugs for initial treatment. Now that multidrug therapy is being implemented, we think it is necessary to examine the effects on platinum sensitivity after using each drug and reconsider the PFI.

Bevacizumab is a monoclonal antibody against vascular endothelial growth factor-A (VEGF-A), which inhibits the binding of VEGF-A to its receptors, causing regression of angiogenesis that supplies oxygen and nutrients to the tumor (Jain, 2014) and induce tumor dormancy (Indraccolo, 2013). Additionally, VEGF induces immune tolerance by reducing the cytotoxic activity of T cells (Ziogas et al., 2012) and promoting regulatory T cell proliferation (Terme et al., 2013). Furthermore, VEGF is known to play a role in forming perivascular niches and promotes self-replication necessary for cancer stem cells (Goel and Mercurio, 2013). Blocking the VEGF pathway can suppress these actions and exert antitumor effects.

When bevacizumab is incorporated with chemotherapy, it is assumed that the vascular normalization effect improves the pharmacodynamics of the chemotherapy drugs, leading to increased chemotherapy sensitivity. In contrast, progression-free survival (PFS) may be extended regardless of the chemotherapy sensitivity of tumor cells owing to mechanisms of action that do not directly modify the chemotherapy sensitivity of tumor cells; these include tumor dormancy from angiogenesis inhibition (Indraccolo, 2013), reactivation of antitumor immunity Ziogas et al., 2012, Terme et al., 2013, Ohm and Carbone, 2001; and involvement of cancer stem cells (Goel and Mercurio, 2013). This may cause a discrepancy between the conventional PFI and sensitivity to subsequent platinum-containing regimens. In other words, when bevacizumab is combined, even if the PFI exceeds 6 months and partial platinum sensitivity is determined, platinum agents may not be effective in actual recurrence chemotherapy. Therefore, the purpose of this study was to retrospectively re-evaluate PFI and sensitivity to subsequent platinum-containing chemotherapy by analyzing cases of recurrence after initial maintenance bevacizumab treatment.

## 2 Materials and methods

For this intergroup study, participants were recruited from local Japanese clinical trial groups: Sankai Gynecology Study Group (SGSG), Kansai Clinical Oncology Group (KCOG), and Tohoku Gynecologic Cancer Unit (TGCU). Ethical approval was obtained from the Medical Ethical Committee of the Kawasaki medical university (ethics number: 3812–02). The nineteen participating institutions collected data after obtaining approval from their ethical committee. Informed consent was obtained in the form of opt-out on the website.

This study included patients with stage III or IV ovarian, fallopian tube, or primary peritoneal cancer with platinum-sensitive first recurrence between November 1, 2013, and December 31, 2019, who received platinum-containing chemotherapy in combination with and maintained with bevacizumab as the initial treatment and were treated with platinum at recurrence. Eligibility criteria included histological confirmation of epithelial ovarian, fallopian tube, or primary peritoneal cancer and confirmation of complete response (CR) or no evidence of disease following initial treatment. Patients with other malignant tumors, those treated with radiation therapy, those with non-epithelial or borderline malignant histology, those who underwent secondary debulking surgery before recurrence, and those who were administered PARP inhibitors as maintenance therapy were excluded. A retrospective study was conducted using medical records of all patients. Data were collected based on age, stage of progression (FIGO 2014), histological type, primary lesion, initial treatment information (date of initiation of treatment, treatment details, date of initial surgery, chemotherapy regimen, number of courses, date of last chemotherapy dose, date of last bevacizumab dose, number of bevacizumab doses, etc.), information at the time of recurrence (date of confirmation of recurrence, site of recurrence, time of recurrence, diagnostic method, date of initiation of recurrence treatment, recurrence chemotherapy regimen, whether maintenance therapy was administered), best response (RECIST v1.1), date of confirmation of progression after recurrence treatment and subsequent treatment initiation date, date of last confirmation of survival, and outcome.

The primary endpoints were the platinum-free period and response rate (CR + PR), plotted as 100 % stacked bar graphs every 3 months for comparison. Additionally, PFI was divided into 6 ≤ PFI < 12, 12 ≤ PFI < 24, and 24 ≤ PFI, and trend analysis of each group and response rate was performed using the Cochran–Armitage test. Furthermore, the influence of the platinum-free period and after-relapse treatment were evaluated as secondary endpoints using PFS from the time of the first relapse. PFS was divided into 6 ≤ PFI < 12, 12 ≤ PFI < 24, and 24 ≤ PFI, respectively, and evaluated using the Kaplan–Meier method, and comparisons between groups were performed using the Log-rank test. The significance level was set at *p* < 0.05. The time from the last bevacizumab administration to the initiation of relapse treatment and the response rate (CR + PR) were plotted as 100 % stacked bar graphs every 3 months, and trend analysis of each period and response rate was performed using the Cochran–Armitage test. The analyses were performed using SPSS for Mac version 27.0 (IBM SPSS, Armonk, NY: IBM Corp.).

## 3 Results

In total, 131 patients were registered, and 54 were excluded because of use of PARP agents at recurrence, no recurrence, platinum-resistance, omission of bevacizumab maintenance, recurrent treatment with non-platinum agents, no recurrence treatment, or being outside of the study period (Fig. S1). The data of 77 patients that met the eligibility criteria were analyzed.

The median patient age was 62 years, and the most common histological type was serous carcinoma (59, 77 % of patients). In our study, all patients consisted of the same ethnic group (East Asians). Table 1 presents details of patients’ backgrounds (Table 1).

**Table 1:** Clinical Characteristics.

Clinical characteristics	n = 77
Age-y, median(range)	62(24–86)
Histologic type –No. (%)	
Serous	59(77 %)
Clear cell	10(13 %)
Endometrioid	3(4 %)
Mixed	1(1 %)
Others	4(5 %)
Stage-No. (%)	
III	42(55 %)
IV	35(45 %)

Clinical stage is evaluated using International Federation of Gynecology and Obstetrics (FIGO) classification. y, years.

Table 2 presents the treatment details of the initial treatments. Neoadjuvant chemotherapy (NAC) was performed in 47 (61 %) patients, and primary debulking surgery was performed in 30 (39 %) patients. The most common initial chemotherapy for patients who underwent NAC was tri-weekly paclitaxel and carboplatin (TC) (32, 41 % patients). Bevacizumab was administered during the NAC stage in seven (9 %) patients. Tri-weekly TC therapy accounted for most adjuvant chemotherapy cases (75, 98 %). The median number of times bevacizumab was administered was 18 (range: 4–41), including adjuvant treatment period (Table 2).

**Table 2:** Initial Treatment.

	n = 77
NAC − No.(%)	
Yes	47(61 %)
No	30(39 %)
NAC regimens − No.(%)	n = 47
TC(DC)	32(68 %)
ddTC	8(17 %)
TC + Bev	7(14 %)
Regimens of adjuvant chemotherapy − No.(%)	n = 77
TC(DC) + Bev	75(98 %)
ddTC + Bev	1(1 %)
weeklyPTX + Bev	1(1 %)
No. of cycles of Bev − median(range)	
total cycles	18(4–41)
maintenance cycles	11(1–38)

NAC, Neoadjuvant chemotherapy; TC, paclitaxel plus carboplatin; DC, docetaxel plus carboplatin; Bev, Bevacizumab; ddTC, dose-dense paclitaxel plus carboplatin PTX, paclitaxel.

Table 3 presents the recurrence data. The median PFI was 12 months (range: 6–43), and the median time from the last bevacizumab administration to the initiation of recurrence treatment was 4 months (range: 0 –29). During bevacizumab maintenance therapy, recurrence occurred in 28 (36 %) patients and after completion in 49 (64 %) patients.

**Table 3:** Treatment for Recurrence.

	n = 77
PFI − months, median(range)	12(6–43)
Bev-TFI − months, median(range)	4 (0–29)
Timing of recurrence − No. (%)	
during Bev maintenance	28(36 %)
after Bev maintenance	49(64 %)
Regimens for first recurrence	
Platinum-based	31(40 %)
Platinum-based + Bev	46(60 %)
Response to treatment for recurrence	
CR	19(25 %)
PR	25(32 %)
SD	15(19 %)
PD	16(21 %)
NE	2(3 %)

PFI, platinum-free interval; Bev, Bevacizumab; Bev-TFI, treatment-free interval since last administration of bevacizumab.

CR, complete response; PR, partial response; SD, stable disease; PD, progression disease; NE, not evaluate.

**Fig. 1:**
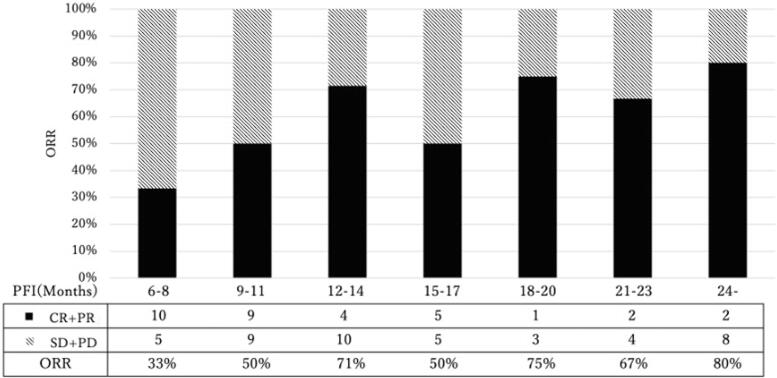
The response rate to recurrence treatment using the PFI divided into 3-month intervals. PFI, platinum-free interval; ORR, overall response rate; CR, complete response; PR, partial response; SD, stable disease; PD, progression disease.

Fig. 1 illustrates the response rate to recurrence treatment using the PFI divided into 3-month intervals (Fig. 1). The overall response rate for 6≦PFI < 12, 12≦PFI < 24, and 24≦PFI was 42 %, 65 %, and 80 %, respectively, showing a linear trend between the duration of PFI and the response rate (*p* < 0.05, Cochran–Armitage test).

**Fig. 2:**
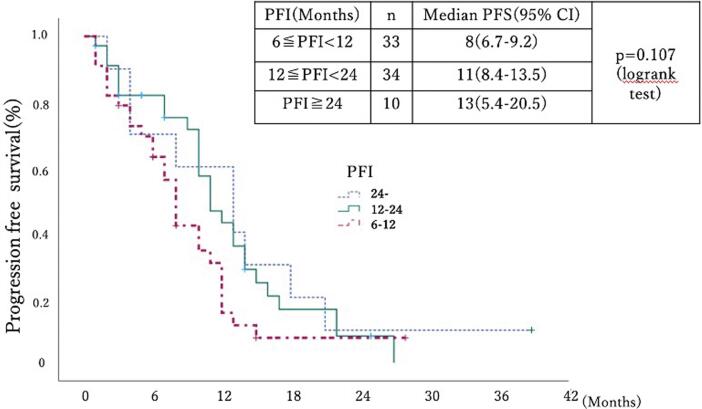
Kaplan-Meier curves of PFS from the first recurrence treatment to subsequent progression or death for each duration of PFI, 6≦PFI < 12, 12≦PFI < 24, and 24≦PFI. PFS, progression-free survival; PFI, platinum-free interval.

The PFS from the first recurrence treatment to subsequent progression or death was evaluated using the Kaplan–Meier method, and the median PFS for 6≦PFI < 12, 12≦PFI < 24, and 24≦PFI was 8 months (95 % CI: 6.7–9.2), 11 months (95 % CI: 8.4–13.5), and 13 months (95 % CI: 5.4–20.5), respectively (*p* = 0.107, log-rank test) (Fig. 2).

**Fig. 3:**
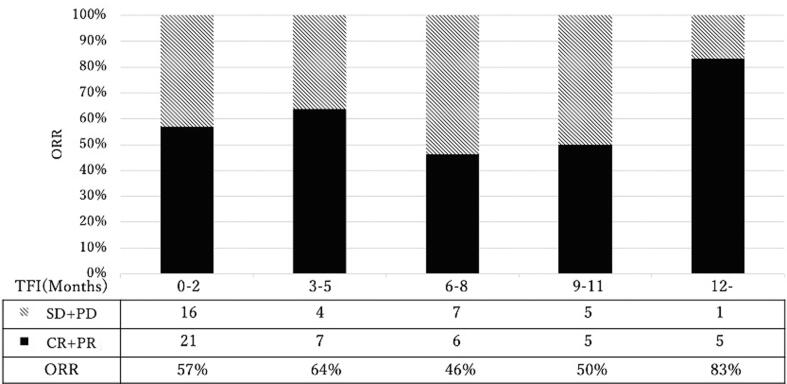
The response rate to chemotherapy at recurrence for each treatment-free interval since last administration of bevacizumab (Bev-TFI). TFI, Treatment-free interval; ORR, overall response rate; CR, complete response; PR, partial response; SD, stable disease; PD, progression disease.

Fig. 3 illustrates the response rate to chemotherapy at recurrence for each treatment-free interval since last administration of bevacizumab (Bev-TFI) (Fig. 3). The response rate was 57 % even when Bev-TFI was 0≦Bev-TFI≦2, which 28/37 (76 %) recurrence occurred during bevacizumab maintenance therapy. Furthermore, no linear trend was observed between Bev-TFI and response rate (*p* = 0.225, Cochran–Armitage test), indicating no correlation between the length of the Bev-TFI and response rate.

When the histological type was divided into serous and non-serous groups, the response rates were 61 % and 43 %, respectively, with no significant difference (*p* = 0.22, chi-square test).

## 4 Discussion

In this study, we investigated the response rate for recurrent treatment when bevacizumab was used as initial treatment in combination with platinum containing regimens, and the overall response rate (ORR) was 42 % in patients with 6≦PFI < 12. When further divided into two groups, the ORR for 6≦PFI < 9 months and 9≦PFI < 12 months was 33 % and 50 %, respectively (Fig. 2). These results indicate that the pharmacological effect of bevacizumab does not seem to alter the sensitivity to subsequent platinum-based chemotherapy.

Regarding the relationship between the efficacy of platinum-based chemotherapy and PFI, Dizon et al. conducted a retrospective study of cases in which TC regimen or single-agent carboplatin was used for recurrent therapy and reported that the response rate was 25 % for TFI 6–12 months and 43 % for TFI > 12 months (Dizon et al., 2003). Additionally, Markman et al. reported that the response rates to platinum agents at recurrence when PFI was less than 12 months, 12–17 months, and 18 months or more were 33 %, 55 %, and 75 %, respectively (Markman et al., 1991). Although it should be noted that the treatment methods in these studies are not completely standardized regarding platinum agents and concomitant drugs used for relapse treatment, Our study results suggest that the response rate is comparable to that of previous reports Dizon et al., 2003, Markman et al., 1991; even in the early period of partial sensitivity (6≦PFI < 9).

We also found that no correlation was observed between the duration of Bev-TFI and response rate to platinum-containing recurrent treatment, and the response rate was maintained even if relapse occurred during bevacizumab maintenance. Several mechanisms of resistance to bevacizumab have been proposed, including up-regulation of alternative pathways such as mediated by angiopoietin, fibroblast growth factor, and platelet-derived growth factor (Rigamonti et al., 2014, Gyanchandani et al., 2013, Crawford et al., 2009), reduced sensitivity to anti-angiogenic therapy owing to increased pericyte coverage around tumor vessels (Thomas et al., 2013); and up-regulation of processes that mimic angiogenesis owing to tumor cells undergoing epithelial-to-mesenchymal transition after bevacizumab (Xu et al., 2012). Although the mechanism of resistance to bevacizumab, including its involvement in the pathway that determines platinum sensitivity, has not been fully elucidated, our study revealed that at least it does not seem to affect platinum sensitivity after relapse. When planning treatment for recurrence after maintenance therapy with bevacizumab, we believe that existing criteria for confirming the indication for platinum agents can be used. In such case, there is evidence for the use of bevacizumab beyond PD (Pignata et al., 2021), and the combination of bevacizumab with cytotoxic agents may be an option.

Currently, PARP inhibitors are commonly used for maintenance therapy after initial treatment. However, resistance mechanisms related to the homologous recombination (HR) pathway such as several reversion mutations in BRCA1/2 and other HR-related genes have been identified after the use of PARP inhibitors (Penson et al., 2022, Lin et al., 2019). It has been reported that PARP inhibitors reduce the efficacy of subsequent platinum-based therapy, in the setting of maintenance therapy after relapse. For example, in a post-hoc analysis of the SOLO-2 study, the time to second progression (TTSP) was significantly longer in the placebo group than in the olaparib group in patients who had received platinum-based chemotherapy, 14.3 months vs. 7.0 months (HR 2.89, 95 % CI 1.73–4.82) (Frenel et al., 2022).

Furthermore, in the OReO/ENGOT-ov38 study (Pujade-Lauraine et al., 2023), which examined re-challenge with PARP inhibitors, PFS was extended regardless of BRCA status, but the eligibility criteria strictly required a specific period from previous PARP inhibitor administration to recurrence. For example, the entry criteria for the BRCA mutation cohort were 18 months or more after first-line recurrence and 12 months or more after second-line recurrence, imposing longer disease control periods than usual. Based on these clinical data, we thought that PARP inhibitors are likely to influence platinum sensitivity after relapse and decided to exclude cases in which PARP inhibitors were used and purely examine the effects of Bevacizumab on PFI.

Recently, combination therapy with cytotoxic drugs and multiple molecular-targeted drugs such as PARP inhibitors and angiogenesis inhibitors has become common in the initial treatment of ovarian cancer, making it more difficult to judge platinum sensitivity based on PFI at recurrence. Further investigation is needed into the effects of molecular targeted drugs on platinum sensitivity and to explore biomarkers that can confirm platinum sensitivity. For example, when combined with PARP inhibitors, clinical applications of reversion mutation using circulating tumor deoxyribonucleic acid (ctDNA) might be promising [26].

Our study has several limitations because of its single-arm, small sample size, retrospective design and use of real-world data. Therefore, there are challenges including issues related to inconsistent data quality, comparability and bias. However, there is substantial data examining the effects of platinum drugs on PFI (Pujade-Lauraine, 2012, Gore et al., 1990, Colombo and Gore, 2007, Blackledge et al., 1989, Raja et al., 2013, Dizon et al., 2003, Markman et al., 1991); and we believe that it is sufficient to complement comparisons with common platinum-containing regimens.

## 5 Conclusion

In this study, when bevacizumab was used in combination with the initial treatment, the response rate was maintained even if the PFI corresponded to the early period of partial sensitivity. Additionally, as no correlation was observed between the duration of Bev-TFI and the treatment response rate with platinum-containing regimens at relapse, applying PFI as a criterion for determining platinum sensitivity is acceptable, even if relapse occurs during bevacizumab maintenance. It is necessary to further investigate the effects of other combined molecular target drugs on platinum sensitivity in subsequent treatments.

## CRediT authorship contribution statement

**Tamaki Tanaka:** Writing – review & editing, Writing – original draft, Visualization, Methodology, Investigation, Formal analysis, Data curation. **Kazuhiro Takehara:** Writing – review & editing, Supervision, Data curation. **Tomoka Usami:** Writing – review & editing, Data curation. **Masako Ishikawa:** Writing – review & editing, Data curation. **Eiji Kondo:** Writing – review & editing, Data curation. **Masahiro Kagabu:** Writing – review & editing, Data curation. **Kei Hirabayashi:** Writing – review & editing, Data curation. **Noriomi Matsumura:** Writing – review & editing, Data curation. **Shinya Sato:** Writing – review & editing, Data curation. **Masato Nishimura:** Writing – review & editing, Data curation. **Atsushi Arakawa:** Writing – review & editing, Data curation. **Keiichiro Nakamura:** Writing – review & editing, Data curation. **Yosuke Konno:** Writing – review & editing, Data curation. **Satoe Fujiwara:** Writing – review & editing, Data curation. **Kotaro Sueoka:** Writing – review & editing, Data curation. **Hiroko Nakamura:** Writing – review & editing, Data curation. **Iemasa Koh:** Writing – review & editing, Data curation. **Kimihiko Ito:** Writing – review & editing, Data curation. **Atsushi Hongo:** Writing – review & editing, Visualization, Supervision, Methodology, Investigation, Formal analysis, Data curation, Conceptualization.

## Declaration of competing interest

The authors declare the following financial interests/personal relationships which may be considered as potential competing interests: [K. Takehara received grant support and honoraria fees from Chugai pharmaceutical Co., Ltd. N. Matsumura received grant support from AstraZeneca plc. and participated in speaker’s bureau sponsored by AstraZeneca plc., Takeda pharmaceutical Co., Ltd., MSD Co., Ltd., Eisai Co., Ltd., Chugai pharmaceutical Co., Ltd. K. Ito received honoraria fees from Chugai pharmaceutical Co., Ltd. They have received these grants for work outside the submitted work. The other authors have no potential conflict of interest to report].
