# Supplementary figures and images for: Evaluation of platinum-free interval and chemotherapeutic effect of subsequent platinum-containing chemotherapy in patients with recurrent ovarian cancer initially treated with bevacizumab: SGSG018/Intergroup study
Source: Gynecol Oncol Rep. 2025 Apr 9;59:101740. doi: 10.1016/j.gore.2025.101740 (PMC12036064; doi:10.1016/j.gore.2025.101740)

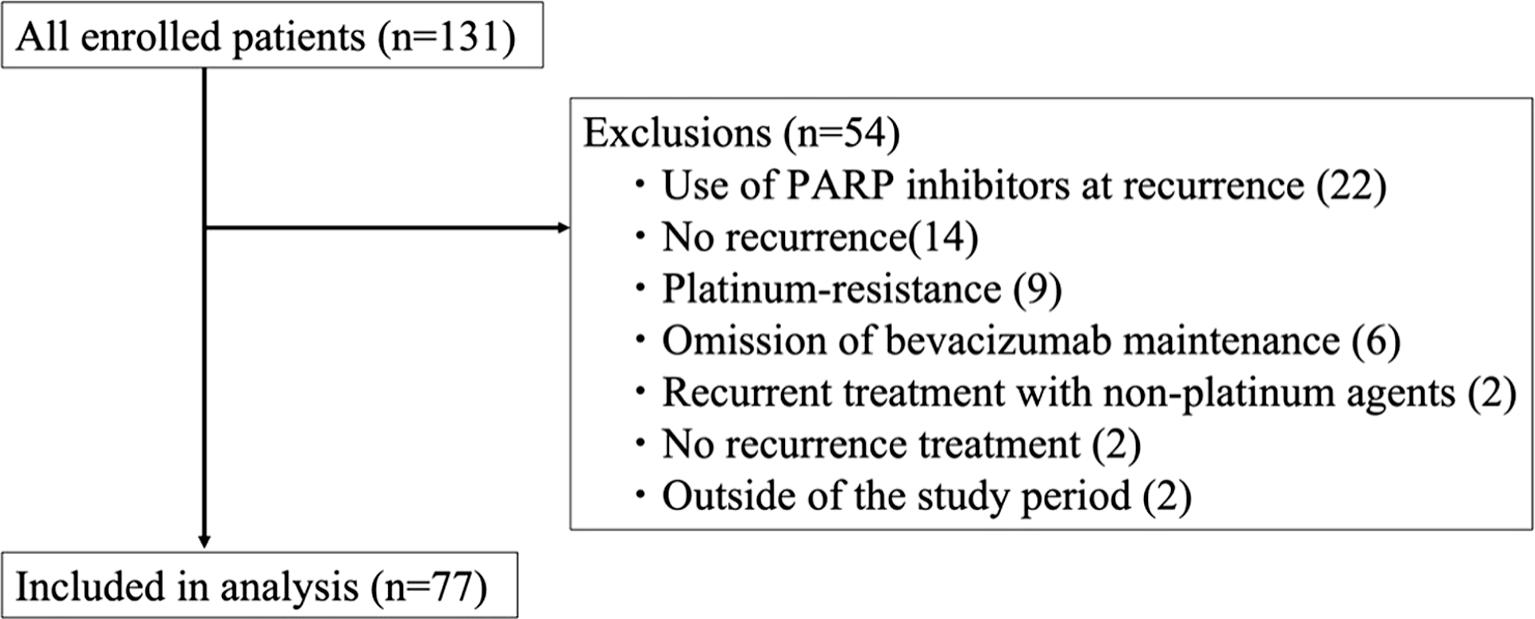

Supplement: Supplementary Figure 1 [file mmc1.jpg]
